# Supplementary material for: Are carbon emissions trading and green financial instruments synergistic? -Comprehensive quantitative research based on content analysis
Source: PLoS One. 2024 Mar 7;19(3):e0298601. doi: 10.1371/journal.pone.0298601 (PMC10919601; doi:10.1371/journal.pone.0298601)
Supplement: S1 Data — (ZIP) [file pone.0298601.s001.zip › Data/地级市Z_F工作报告.zip!/║■▒▒/╦μ╓▌╩╨/└┤╫╘╣1⁄2╓┌║┼í╛┬φ┐╦╩2╛▌═°í┐_2010.docx]

各位代表：

现在，我代表市人民政府向大会报告政府工作，请予审议，并请市政协委员和各位列席人员提出意见。

一、2009年工作回顾

刚刚过去的2009年，是我市在复杂宏观背景下各项工作都取得突破性进展的一年。面对异常严峻的经济形势，市政府紧紧依靠全市人民，深入贯彻落实党的十七届三中、四中全会精神，扎实开展学习实践科学发展观活动，正视风险，保持清醒，坚定信心，积极应对，抢抓中部崛起和湖北“两圈”建设机遇，化危机之年为赶超之年，全市经济保持了超过年初预期、高于全国和全省平均水平的 “一超二高”良好发展态势，各项社会事业全面进步，圆满完成了市二届人大四次会议确定的目标任务。

——综合实力显著提高。2009年，全市实现地区生产总值344.14亿元，比上年增长13.5%，其中一、二、三产业分别增长6%、14.5%和17%。全年实现财政总收入17.45亿元、同比增长19.1%，其中地方一般预算收入7.6亿元、增长17.4%。

——“三驾马车”强劲驱动。投资、出口、消费“三驾马车”一起发力，发展的外部拉力和内生活力明显增强。全年完成全社会固定资产投资208.7亿元，同比增长41.9%，投资对经济增长的贡献率达到40%以上。实现外贸出口4.65亿美元，增长38.1%。消费市场全面激活，社会消费品零售总额182亿元，增长19.5%。

——人民生活进一步改善。农村人均纯收入5457元，比上年净增490元，增长9.9%；城镇居民人均可支配收入13461元，增长16.1%。社会保障体系不断健全，城镇新增就业1.8万人，城乡居民养老、医疗、失业、工伤和生育保险等保障水平继续提高。

过去一年，我们全力以赴保增长，坚持不懈保民生，坚定不移保稳定，许多工作有了新的突破：

（一）以工业兴市为主导，经济实力进一步提升。按照保增长重点保工业的要求，积极引导企业抱团取暖、差异化发展，在困境中寻找商机，建立了政府对企业一企一策、对口帮扶的服务机制，帮助企业解决了150多个突出困难和问题，促使工业经济保持了较快增长，实现规模工业总产值350亿元，增长23.9%；支柱产业快速扩张，五大支柱产业实现增加值75.7亿元，增长20.1%，规模工业增加值、汽车机械总产值双双突破百亿元大关。狠抓中小企业成长工程，全市净增规模工业企业40家、总数达到549家，其中亿元企业69家。实施精品名牌战略，“齐星”、“大力”荣获中国驰名商标，实现零的突破。县域工业快速发展，成为拉动全市工业增长的重要力量。

（二）以新农村建设为着力点，城乡发展进一步协调。积极应对连续干旱和病虫害的袭击，农村经济取得了“三增”的好成绩，粮油连续7年增产、农业效益持续增长、农民连续10年增收。粮食总产达到150万吨，同比增长2.4%；棉花、油料分别增长2.6%和8.5%；10万农民工集中返乡的矛盾得到有效缓解。进一步调整农业内部结构，高效农业、创汇农业较快发展，食用菌、马铃薯、菜豌豆、优质桃和小龙虾等特色板块增效增收，随县草店镇被授予“中国花菇之乡”。加快农业产业化发展步伐，“一袋面”工程进展顺利，新增省级龙头企业2家，组建各类专业合作社50个，新建村级综合服务社100个，社会化服务体系不断完善。加快农村基础设施建设，“一建三改”全面推进，建成沼气池1.5万口，改厕1.45万户；新建通村水泥路1260公里，行政村通车率达到97%。随南和广水北三镇新农村试验区建设稳步推进，高标准完成了160个村的村庄整治任务，25个村“整村推进”扶贫工作进展顺利。加快建设林业生态强市和林业产业大市，完成低产林改造和各类造林10万亩。开工各类水利工程1.2万处，解决了16.5万农村人口的饮水安全问题，水库除险加固、灌区续建与节水改造进展顺利，农田水利基本建设得到加强。

（三）以项目建设为载体，投资规模进一步壮大。把项目建设作为撬动经济增长的“黄金支点”，深入开展“大项目年”活动，继2007年固定资产投资过百亿元之后，去年又突破200亿元大关，其中城镇以上投资165.8亿元，同比增长45.9%。对上争资工作取得突破性进展，全年争取无偿资金4.4亿元，36个过亿元项目开工，武康铁路增建二线随州段、随岳北高速随州段、长岭高架桥建成通车，西气东输随州段顺利开工，麻竹高速东段、广水污水处理厂等按计划推进；28个市级重点项目建设进展顺利，齐星多晶硅项目克服重重困难，即将投入试运行。

（四）以深化改革、扩大开放为突破口，发展活力进一步显现。创新招商思路，拓展招商领域，突出招商重点，组织参加了鄂京环渤海湾地区经贸洽谈会、第四届中博会等一系列重大招商活动，全年共引进2000万元以上的项目117个，实际利用内联资金45.5亿元，同比增长12%；实际利用外资4402万美元，同比增长13%，在建亿元引资项目达到13个。大力实施外向带动战略，全面落实促进外贸出口的政策措施，扶持出口主体，拓展新兴市场，扩大对外贸易，实现了以农副产品为主体向农副产品、汽车机械、电子信息、轻工服装多元化发展的转变，我市成为全国拥有整车出口资质最多的城市，在全国、全省外贸走跌的大背景下，我市对外贸易逆势上扬，增幅高于全省平均水平53个百分点。以产权制度改革为核心，实施国企改革攻坚，省专汽进入破产程序，铁树资产回购、出让及遗留问题处理有序推进，中国恒天重组新楚风工作进展顺利；非工业企业改革步伐加快，国有粮食企业改革经验在全国推广，集体林权制度改革完成率达到94%；政府机构改革、人事制度改革按计划推进。成立新的随县和曾都区，构筑起区域经济 “三足鼎立”、竞相发展的新格局。经济开发区“三通一平”快速推进，发展承载力不断提升。

（五）以加强银企合作为切入点，金融生态环境进一步优化。注重发挥金融助推经济发展的引擎功能，大力推进政企、银企对接合作，引导企业规范财务管理，降低融资成本，积极争取银行信贷支持；鼓励金融机构创新信贷产品，加大投放力度，满足各类企业特别是中小企业的合理资金需求。金融机构新增贷款的总量和增幅实现历史性突破，到12月底，全市新增贷款43亿元、比年初增长53.1%。出台优惠政策，对下岗再就业、自主创业给予信贷支持，累计发放财政贴息小额担保贷款4400万元。保险事业平稳较快发展，实现保费收入11.3亿元、同比增长17.5%。金融生态环境进一步优化，我市被授予全省“A级金融信用市”，广水市、曾都区被授予“最佳金融信用区”。

（六）以提升旅游竞争力为重点，第三产业进一步繁荣。围绕擦亮两张文化名片，打造“鄂西圈”重要的旅游目的地和集散地，高质量编制完善旅游规划，高规格举办首届世界华人炎帝故里寻根节、元旦拜祖祈福盛典等系列活动，全面提振了城市的人气、品牌的名气和干群的士气；同时通过发挥办节的“倒逼机制”，促进了政府系统工作效率和工作水平的提高。突破性推进景区景点和旅游项目建设，炎帝故里、玉龙温泉当年建成、当年双双跻身国家4A级景区；一批星级宾馆建成使用，旅游接待条件明显改善，一批镇、村被评为全省旅游名镇、旅游名村，创建“中国优秀旅游城市”工作顺利通过省初检评审。全年实现旅游总收入26亿元，同比增长40%。千方百计扩大市民即期消费需求，不断加快城市社区便民商贸服务设施建设，继续实施“万村千乡市场工程”和“双百市场工程”，发放家电下乡补贴2750万元，建成农家店372家、2个大型配送中心；高度重视成品油、农资、卷烟、生猪屠宰等专业市场的管理整顿，商品质量、价格监测网络初步建成，城乡市场秩序进一步规范。

（七）以加强建管为抓手，城市形象进一步改善。按照“城市建设一天不停步、城市管理一刻不放松”的要求，通过经营城市资产、积极对上争取、加大财政投入等多种渠道，筹措资金近10亿元，开工建设一大批城市功能项目。神农大道、白云湖大坝和滨湖风光带配套完善，白云公园、滨湖体育公园、明珠广场改造顺利完成；新火车站及配套工程如期投入使用，迎宾大道延伸和白云大道二期建成通车，波导大桥开工建设，316国道改造全面启动，城东新区路网建设基础性工程基本完成。大面积刷黑城市主干道，新增、改造城区绿地60多万平方米，成功创建“湖北省园林城市”。坚持以人为本、建管为民的发展理念，依法行政，文明执法，构建了防违拆违、门前四包、条块结合、社区为主的城市管理长效机制，推进了城市规模与管理层次的同步提升，城市品位与市民素质的同步提升。加大“楚天杯”、“神农杯”创建力度，着力提高中心镇的规划和建管水平。加强建筑市场监管，房地产业实现了理性化发展。

（八）以改善民生为立足点，和谐随州建设进一步推进。始终不渝地把“保民生”作为政府工作的立足点和落脚点，先后出台两个含金量较高、支持全民创业的政策性文件，积极推动创业带动就业和回归创业两大工程，开发300个公益性就业岗位，安置城镇“零就业家庭”和特困人员就业，全市新增市场主体近万户、日均26户。五项基本保险扩面新增5.1万人，参保总数达到68.5万人，辞退民师养老保险基本实现全覆盖；再次上调养老金，人均增加102元；新型农村合作医疗参保率达到94.2%，累计发放住院补贴近2亿元。强化宗旨为民解难，不断加大社会贫困群体救济保障力度，累计发放各类救济救灾钱物1680万元；不断加大扶贫开发力度，减少贫困人口1.1万人。扎实推进住房保障工作，加快安居工程建设，南水北调库区移民安置点建设全面启动。年初市政府承诺的十件实事全部完成或超额完成。积极落实义务教育经费保障政策，提高寄宿制困难学生补助标准，改造农村薄弱学校73所；加快推进素质教育，高考万人上线率位居全省前列，职业教育进一步提档升级。加快科技创新步伐，成功申报国家和省级科技计划项目28项，高新技术产业增加值同比增长11%。实施文化惠民工程，新建和改造乡镇综合文化站（馆）10个，建设“农家书屋”459家，完成20户以上自然村有线电视“村村通”128个。广泛开展群众性文化体育活动，全民健身运动蓬勃开展，市博物馆、图书馆免费接待市民及游客90多万人次，网吧监管得到加强。公共卫生服务体系不断健全，医药卫生体制改革全面启动，医疗质量稳步提高；及时果断处置了手足口病、甲型H1N1流感、人粒细胞无形体病等一批突发公共事件，公共卫生应急能力不断提高。重视和加强人口与计划生育工作，符合政策生育率达到94.28%、高于省定目标2.3个百分点，出生人口性别比较上年降低2.44，基层计生服务站经验在全省推广。坚持走科学发展、集约发展之路，落实最严格的耕地保护和土地供应制度。狠抓节能减排，全面完成单位生产总值能耗、二氧化硫和化学需氧量约束性指标。坚持打防并举，健全社会治安防控体系，确保了新中国成立60周年、寻根节等重大节庆活动期间秩序良好，人民群众的安全感和满意度得到提升。深入开展依法治市，稳步推进“五五”普法。进一步加强信访工作，坚持以人为本，注重工作的方式方法，避免在加快发展的过程中，特别是在征地拆迁等敏感问题上产生新的矛盾。安全生产形势继续保持总体稳定，各类事故总量大幅度下降。加强国防和民兵预备役工作，军政军民关系进一步密切。

（九）以问责纠偏为手段，政府自身建设进一步加强。

一年来，我们围绕建设服务型政府，全面履行职责，加快转变职能，政务环境不断改善。推进依法行政，认真执行市人大及其常委会的决议，自觉接受人大法律监督和工作监督，支持人民政协履行职能，144件建议提案办结率、满意率都达到100%。注重加强同民主党派、工商联、无党派人士、人民团体和社会各界的联系，认真听取他们的意见和建议。修订完善政府工作规则，行政决策机制进一步健全。围绕打造全省发展成本最低、行政审批事项最少、行政服务效率最高的城市，以“作风建设年”和“能力建设年”为主题，全面推行首问负责制、限时办结制和问责追究制，扎实开展民主评议政风行风活动，实施政府绩效评估，强化政务督查；进一步简化办事程序，精简审批项目，开通电子政务，推进行政提速。加强勤政廉政建设，严格落实党风廉政建设责任制，加大对财政专项资金、民生工程等领域的审计力度，大力提倡勤俭节约，政府性资金的管理和使用更加规范。

人民防空、工商、统计、国调、妇女儿童、老龄工作得到加强，对台、气象、档案、地方志以及民族宗教、外事侨务、食品药品监督、质量技术监督、供电、移动通信、无线电管理等工作都取得可喜成绩。

过去一年，我们经受了严峻考验，取得了应对危机重要的阶段性成果。成绩的取得极其不易、弥足珍贵，这是各种政策效应综合释放、相互支撑的结果，更得益于市委的科学谋划、措施有力；得益于市人大、市政协的有效监督、支持帮助，得益于各级各部门的密切配合、尽职尽责；得益于全市人民的共克时艰、拼搏进取。在此，我代表市人民政府，向各位人大代表、政协委员，向全市各条战线上辛勤工作的广大干部群众，向驻随部队、武警官兵和政法干警，向各民主党派、工商联、人民团体和驻随单位，向所有关心支持随州发展的老领导、同志们、朋友们表示衷心的感谢，并致以崇高的敬意！

我们也清醒地看到，政府工作还有不少薄弱环节和问题，经济社会生活中存在不少突出矛盾。主要表现在：一是三次产业占比不优，结构性矛盾仍然突出。农业比重偏大，工业支撑不强，财政规模较小，需要下大力气并通过一段时间坚持不懈地努力，来加以调整和优化。二是多点支撑的产业格局尚未形成。五大支柱产业发展不够平衡，在专汽产业加快发展的同时，医药化工、电子信息、纺织服装、食品加工等产业的支柱作用有待进一步提升。三是赶超发展的压力进一步增大。同类城市发展势头咄咄逼人， “不进则退、慢进亦退”的现实给我们提出巨大挑战。四是社会事业欠账较多，维护稳定压力较大。社会保障、教育、医疗等涉及人民群众切身利益的不少突出问题还没有根本缓解；随着改革发展进入攻坚阶段，统筹各方面利益的难度增大。五是政府自身建设有待加强。极少数部门和干部思想不够解放、大局观念不强、服务意识淡薄、执行力不够等问题还客观存在，一些制度上还有缺失、管理上还有漏洞、监督上还有缺位。对于这些矛盾和问题，我们将在今后的工作中认真加以解决。

二、2010年政府工作的形势和任务

2010年是随州建市十周年，是执行“十一五”规划的收尾年，也是谋划“十二五”发展的基础年。做好今年的各项政府工作，具有特别重要的意义。2010年政府工作的指导思想是：全面贯彻党的十七大和十七届三中、四中全会精神，紧紧围绕中央、全省和全市经济工作会议部署，坚持以科学发展观统领全局，按照“加快发展上速度、跨越发展上规模、科学发展上水平”的总体思路，坚定不移地实施工业强市战略，坚定不移地把招商引资作为助推经济发展的主动力，坚定不移地推进城镇化和新农村建设，坚定不移地加快发展各项社会事业，突出结构调整，大抓项目建设，统筹城乡发展，更加注重民生，确保主要经济指标增幅高于全省平均水平，政治、经济、文化和社会建设协调推进，努力开创“四个随州”建设新局面。

今年经济和社会发展的主要预期目标是：地区生产总值增长12%；规模工业增加值增长16%；全社会固定资产投资增长26%以上；地方一般预算收入增长12%；外贸出口增长10%；城镇居民人均可支配收入增长9%，农民人均纯收入增长7%；社会消费品零售总额增长17%；居民消费价格涨幅控制在4%以内；单位生产总值能耗下降4%，二氧化硫和化学需氧量排放总量超额完成“十一五”规划确定的削减目标。各项社会事业全面协调发展。

上述目标是一个预期目标。在实际工作中，我们将自加压力，自压担子，按照“跳起来摘桃子”的要求和科学理性的原则，促使各级政府及其部门以更加积极进取的姿态，确定更高的工作目标，并以工作目标来保证预期目标的全面完成，争取更好的发展结果。做好今年的工作，我们将牢牢把握以下原则：

——更加注重科学发展。坚持“好”与“快”相结合，把更好作为方向，把更快作为前提，始终抓住第一要务，转变发展方式，破解发展难题，坚持不懈做大经济规模；坚持“应急”与“谋远”相结合，更加注重发展的可持续性，促进速度与结构、质量、效益相统一。

——更加注重项目主导。坚持把扩大投资作为富民强市的重要手段，作为夯实基础、蓄积后劲、促进可持续发展的长久之计。实施项目带动，把握导向，改善结构，确保投资总量较快增长。

——更加注重彰显特色。坚持以特取胜、扬长避短、借势发展，打造“两圈”节点和“西圈门户”，擦亮两张名片，建好专汽之都，做强特色农业，提升综合竞争力。

——更加注重改革创新。坚持把加快形成有利于科学发展的体制机制作为最根本、最有效、最持久的促进因素，解放思想、拓宽思路、大胆试验，在推进全民创业、培育消费热点、激活社会资本等方面拿出新举措，为发展增添强劲动力和旺盛活力。

——更加注重以人为本。坚持把促进社会公平正义作为政府执政的核心价值，突出发展的普惠性和公共服务的均等性，充分尊重群众意愿，把新增财力更多地投向民生领域，投向公益事业，投向困难群众和弱势群体，努力让全市人民得到更多实惠；同时，注重化解前进中的各种矛盾，避免产生新的不和谐因素，促进形成干事创业的良好氛围。

今年的发展目标是在深刻分析我们目前的处境、充分考虑随州发展的需要和可能后确立的。一方面，我们清醒地看到了当前所面临的复杂形势。就大背景而言，国家和省有关方面一致认为，如果说2009年是进入新世纪以来我国经济发展最为困难的一年，那么今年将是最为复杂的一年。尽管世界经济有回暖迹象，我国在全球率先实现经济形势总体回升向好，但经济企稳回升，并不意味着经济全面复苏，并不等同于经济运行根本好转。中央和全省经济工作会议指出，今年世界经济运行存在诸多变数，不排除金融风险再次冲击实体经济的可能。就我市而言，在全国全省外贸形势短期内难以根本好转的宏观背景下，继续保持我市出口高位运行的增长态势压力较大；地方财力比较薄弱，在国家进一步强化地方配套约束的条件下，对上争资的压力增大，解决民生问题的能力不足、回旋余地偏小；尽管国家继续实施适度宽松的货币政策，但信贷规模将相对缩小，由去年的10万亿缩减为7.5万亿左右，加之我市企业法人治理结构不够完善，与金融机构之间信息不对称，增加授信和贷款额度将面临新的考验。另一方面，我们也欣喜地看到了拥有的各种有利机遇。国家继续实行积极的财政政策，继续扩大内需、力保外贸出口份额，对于我市专用汽车、旅游、房地产、外贸出口等行业保持平稳增长提供了可能；随着城市功能的完善和城市形象的提升，承接沿海产业转移和招商引资的势头正在往好的方向发展，如果我们引导得当、服务到位，一大批国有大型企业和沿海企业将沿着了解随州、走进随州、直至投资随州的 “三部曲”来推进；经过改革开放的充分洗礼，特别是建市10年的发展，我们积累了丰富的发展经验，培育了优秀的干部和企业家队伍，随州的地缘人缘商缘优势仍在，产业配套优势仍在，项目发展前景仍在，市场基本需求仍在，为跨越发展奠定了坚实基础。同时，处于赶超发展的关键时期要求我们必须主动作为。船行中流，考验我们的耐力和韧性，挑战我们的智慧和勇气。我们必将进一步增强忧患意识和加快发展的紧迫感，紧紧围绕走在全省同类城市前列的奋斗目标，深刻认识市情，找准发展定位，自觉融入国家宏观发展战略，深度参与湖北“两圈”和“襄十随”城市群建设，坚定信心不动摇、奋力赶超不松劲、创新求变不停步、远谋近施不懈怠，借势而为，顺势而动，努力把改革发展稳定各项工作做得更好。

三、2010年政府工作重点

围绕今年目标任务，将着力抓好以下重点工作。

（一）突出工业兴市，调整优化结构。坚持优先发展工业的指导思想不动摇，按照市委提出的“结构调整年”的要求，以实施“六个五十”计划为抓手，即抓好50家骨干企业、培育50家“小进规”企业、新上50个过5000万元的项目、完成50个重点技改项目、加快50个重点高新技术产品的产业化和推广应用、争取50个工业项目获得国家和省资金支持，以增量结构的调整带动存量结构的调整，不断优化产业结构、产品结构、行业结构和企业组织结构。围绕推进“五大工程”，努力构建具有鲜明特色和竞争优势的随州工业格局。

一是强力推进骨干企业巨人工程。把培育壮大骨干企业作为经济发展的重中之重，继续实行一企一策、对口帮扶，选择50家成长性较好的企业进行重点扶持，加速对外合作重组，努力培育一批过10亿元、50亿元的企业。以更大的力度推进企业上市，滚动培育一批上市后备企业，力争今年有1-2家进入上市辅导期。

二是强力推进产业集群升级工程。围绕五大支柱产业的提档升级，引进一批关联企业和重点项目，加速技术改造，拉长产业链条，形成上下工序配套、分工明确、功能齐全、附加值高的产业集群，力争在跻身全省“千亿元产业”和“支柱产业倍增计划”中有所作为。突出抓好汽车及零部件产业发展，进一步整合资源，加强联合，着力解决产品趋同、质量低劣和恶性竞争等问题，调整经营策略，谋求差异化发展；进一步提升产品质量，加强售后服务，探索在重点销售地区建立随州专用车服务网点，全力打造中国最大的专用汽车生产基地和铸件生产基地，全省重要的车桥、风机、模具生产基地；加快三环与楚威战略重组步伐，全力支持恒天新楚风整车项目的建设，进一步完善汽车产业链条，提升“中国专用汽车之都”的竞争力。加大铁树纺织项目二次招商力度，着力打造一家10-20万锭生产能力的龙头企业，带动随州纺织服装产业振兴。尽快解决爱多工业园遗留问题，盘活存量资产，深化与国家级自主创新示范区东湖高新区的合作，推动我市电子信息产业加快发展。进一步支持多晶硅项目建设，积极引进关联企业，加快发展光伏产业。

三是强力推进中小企业成长工程。深入开展“市场主体增量”行动，全面落实促进中小企业发展的各项政策性措施，优化帮扶机制，加大资金投入，引导“草根经济”加速低成本扩张，壮大随州中小企业群体，确保今年净增规模以上工业企业50家以上。

四是强力推进自主创新工程。大力推进质量兴市、兴企战略，加大引智力度，扶持企业技改创新和扩能改造，突出抓好犇星公司热稳定剂、江南消防车、双剑鼓风机、楚胜半挂车等一批重点技改项目，淘汰落后设备、工艺和技术，建成5-10个产学研共同体或技术研发中心，促进企业提高自主创新能力。重点抓好高新技术产品的产业化和推广应用，在政府采购、产品推广、项目安排等方面给予倾斜，提高市场占有率。

五是强力推进园区建设工程。坚持统一规划、分步实施、稳步推进，加快城东新区10平方公里、两水开发区8平方公里的基础设施建设，实现开发区平台建设由线到面的扩张，拓宽新的发展空间。大力实施“满园工程”，探索园区整体包装招商新方式，力争市经济开发区综合实力在全省位次前移。继续支持区市县以及乡镇工业园建设，鼓励企业向园区聚集，推进人才、劳务、技术和资金等生产要素向园区流动，打造经济发展的重要增长极。

（二）强化项目支撑，保持投资增势。继续开展“大项目年”活动，把对上争资和项目建设作为贯穿全年的重大举措，力争今年实现全社会固定资产投资280亿元以上，为更好更快地发展增添后劲。在项目的争取上，密切关注政策动向，精心筛选、包装项目，做好项目前期工作，加强沟通对接，力争全年对上争取无偿投资不少于4亿元。在项目的建设上，坚持突出重点的原则、需要与可能的原则，保证重大项目建设有序接替、再掀高潮。一方面，加快麻竹高速东段、西气东输随州段、波导大桥等一批已开工项目的建设进度，使之尽早建成、产生效益；另一方面，积极创造条件，力争随信铁路、316国道十岗至厉山一级公路等一批新建项目早日动工；同时，结合“十二五”规划编制，抓紧储备麻竹高速西段等一批事关随州长远发展的项目，确保投资持续、稳定、较快增长。在项目的管理上，进一步完善项目推进工作机制，为重点项目提供一条龙、无缝化和“保姆式”服务；加强和改进投资管理，保证资金的高效安全使用，努力建设优质工程、廉洁工程。

（三）围绕增效增收，加快新农村建设。始终把“三农”工作作为重中之重，以贯彻落实中央1号文件各项惠民政策为契机，坚持以工促农、以城带乡，按照“主攻项目、奋战‘两区’、保护民利、提质增速”的工作思路，确保农业增加值增长6%、农民人均增收380元以上，为全市经济回升向好提供基础支撑。

调整结构，壮大特色农业规模。深入推进农业结构调整，继续抓好粮食优势产业，着力提高综合生产能力，确保今年粮食总产稳中有升。加快发展食用菌创汇产业，推进资源转换和品种转型，稳步发展木腐菌，突破性发展草腐菌；大力发展马铃薯、菜豌豆、优质桃、油茶、核桃和小龙虾等高效板块农业，建成10万亩高产油茶和核桃林基地；按照规模化、标准化的要求，建好养殖小区，积极创建畜牧大县、畜牧大镇和全国优质商品猪、肉牛生产基地。加快重点防护林、生态公益林和绿色家园示范工程建设，加大保护和恢复森林植被力度，力争3至5年再造一个绿色随州。

奋战“两区”，加快农业产业化经营。继续推进随南、广水北三镇新农村试验区建设，依托资源优势和产业特点，按照一村一品、一乡一品的思路，完善试验区各镇、村发展规划，连点成线，连线扩面，逐步推开，使之成为集特色农业发展、现代农业开发、农民增收、农田水利基本建设、农村环境改善于一体的新农村建设先行区、样板区；加快推进农产品物流园区建设，使之成为集粮棉油深加工、生猪屠宰等为一体的标准化产业园。大力支持同星农业、中盐银港、华谊油茶、联创核桃等龙头企业发展，推进关联企业联合协作，使产业、生态和龙头企业三方良性互动。

强化服务，促进农业提质增效。统筹安排、打捆使用各类涉农项目资金，重点扶持“新网工程”和种养基地建设。鼓励组建各类专业合作社，抓好农民经纪人和农村实用人才培训，加快农业科技成果转化，提高农民科学种养水平。切实加强对农村资产、资金、资源的清理和监管，探索村集体经济发展的新路子，力争今年三分之一的村集体经济收入达到5万元以上。

强基固本，改善农村基础条件。继续实施水库除险加固工程，大力开展农田水利基本建设，再解决37.6万农村人口饮水安全问题。加大土地整理和复垦开发力度，建设高标准农田6.4万亩。继续推进“一建三改”工程，新建户用沼气池1万口以上。全面完成南水北调中线工程移民安置任务，继续开展驻村扶贫活动，力争解决1万贫困人口的脱贫问题。

（四）建设旅游强市，促进消费增长。一方面，促进形成大旅游的格局。围绕打造“鄂西圈”的门户城市，突破性加快重点景区景点建设。加强与“西投”公司、三特公司的合作，启动以慈恩寺为核心的大洪山景区开发建设，力争主要景点尽早开工；启动炎帝大像景区和炎帝神农景区二期规划建设，努力办好第二届炎帝故里寻根节；将曾侯乙墓遗址和市博物馆整体规划、完善建设、协调管理，力争尽快进入国家4A级景区；推进玉龙温泉、千年银杏谷、桐柏山太白顶、徐家河度假区、三潭和中华山景区建设。巩固“中国优秀旅游城市”创建成果，力争今年创建2个4A级景区和一批旅游名镇、旅游名村。多层面开展旅游促销活动，广泛推介以两张名片为核心的随州旅游；加大对旅行社、旅游纪念品生产企业的扶持力度，带动旅游产业全面发展。另一方面，努力提升第三产业比重。进一步完善流通体系，加快城乡便民商贸服务设施建设，大力推进“便利消费进社区、便民服务进家庭”的“双进工程”，继续开展家电、农机、汽车和摩托车下乡活动，扩大城乡消费需求。规范发展中介服务组织，加快发展金融保险、信息服务、科技咨询、服务外包等现代服务业，鼓励引导个性化、时尚化、品牌化消费。优化商品房供应结构，培育发展二手房和房屋租赁市场，引导居民合理的住房消费需求，促进房地产业健康发展。整顿规范市场经营和价格管理秩序，加强食品、药品、成品油、卷烟、农资等专业市场监管，为市民营造安全、放心、经济、便利的消费环境。

（五）推进城镇化进程，提升城市形象。坚持把推进城镇化进程作为改善环境、拉动内需、促进经济增长的重要载体和持久动力。

一是长短结合抓规划。立足当前，着眼长远，在新一轮城市总体规划的框架下，抓紧编制城市新区控制性详细规划，尽快完善工业园区、综合交通等各类专项规划，突出抓好城市保障性安居工程和城郊居民点编制工作，力争用3年时间完成全市村镇规划编制任务。

二是完善功能促互通。以拉开新区骨架、完善城市功能为重点，加快城市基础设施建设，力求基础设施早改善、城市价值快提升的双重功效。按照拓展铁东新区、完善城南新区、提升老城区的发展思路，促进三区互通、功能互补、协调互动，力争用3-5年时间，使建成区面积由目前的45平方公里发展到80平方公里左右，真正实现“再造一个新随州城”的宏伟目标。今年，城东新区，重点是加强路网建设，新建新火车站站前大道、编钟大道，改造交通大道，打通青年东路，形成三纵三横的交通网络。城南新区，重点是续建迎宾大道、白云大道、神农大道和滨湖体育公园、森林公园，建成从氵厥水二桥至波导大桥16公里的“一河两岸”循环生态走廊，启动南郊污水处理厂和垃圾处理厂建设。老城区，重点是启动原火车站旧址改造；包装氵厥水河西大堤，全面完成舜井大道、烈山大道、解放路、清河路和白云湖东大堤等5条主干道刷黑工程；加快实施绿化美化工程，努力打造山水生态园林城市。

三是健全机制强管理。着力解决“重建轻管”的问题，强化“门前四包”责任制，加快推进城市管理综合执法，下大力整治乱停乱靠、乱丢乱放、乱搭乱建现象；坚决遏制各类违法违规建设，既做到逢违必拆、逢障必清，又做到依法管理、人性关怀。强化街道办事处和社区居委会城市管理职能，提高城市精细化管理水平。实行市区联动，加强社区办公及服务设施的规范化、标准化建设。

四是城乡联动促一体。支持随县县城建设，支持广水城市建设向新区拓展。按照人口、项目、产业逐步向城镇集中的要求，因地制宜加快推进小城镇建设，择优培育和发展一批特色中心集镇，提高全市城镇化水平；启动第二批“百镇千村”示范工程建设，力争更多的镇、村进入省级试点；继续开展“楚天杯”和“神农杯”创建活动，完成200个村的整治建设任务。

（六）扩大对外开放，增强发展活力。坚定不移地实施开放先导战略，为经济增长破解难题、增添活力。

提高招商质效，着力引进大项目。紧盯大集团、大企业、大项目，进一步拓宽招商思路，继续推行专班招商、联络处驻点招商，精心策划和组织参加重大招商活动；积极推动以商引商，促进形成“引进一个，带来一批，兴旺一业”的连锁效应。注重提高招商专业化水平，建立完善引进项目的筛选甄别机制，去伪存真，去劣存优；突出政策的针对性、灵活性，不断强化“先失后得，让利互惠”意识，加强宣传引导和考核激励，调动各地各部门和广大干部群众的主动性、积极性，营造全民关注招商、支持招商、参与招商的良好氛围，力争今年引进亿元项目不少于5个，引进内联资金50亿元。更加注重对已落地项目的协调服务，提高项目的履约率、到资率、开工投产率，提高客商在随州的满意度。

加强出口主体建设，努力扩大对外贸易。实施以质取胜、精品名牌和科技兴贸战略，大力扶持出口龙头企业发展，重点扶持食用菌、机电、服装等出口企业做大做强，叫响一批随州出口产品原产地品牌。建立贸易摩擦预警和应对机制，提高企业抗风险能力。继续巩固亚洲传统市场，进一步拓展东欧及非洲、南美洲等新兴出口市场，加快承接沿海城市和武汉市的加工贸易产业转移，推进我市外贸发展方式的转变，力争今年出口过千万美元企业至少达到12家。

深化各项改革，致力创新体制机制。继续深化国有工业企业改革，尽快终结湖北专汽、铁树集团改制程序；加快粮食、交通等非工业领域国有企业改革进程，引导建立现代企业制度。在完成政府机构改革任务的同时，稳步推进公共事业市场化改革。加快医药卫生体制改革进程，在缓解群众“看病难、看病贵”问题上真正见到实效。抓好集体林权制度主体改革收尾工作，启动国有林场改革，推动森林、林木和林地使用权的合理流转。

（七）狠抓财税金融，提高保障水平。加强财源建设，严格依法治税，做到应收尽收。硬化预算约束，优化支出结构，大力压缩行政事业经费和一般性支出，突出保运转、保民生、保重点项目，提高资金使用效益。加强镇、村两级债务监管，严格防范债务风险，逐步化解村级债务。加大资源整合力度，引导担保机构加强制度建设，多渠道充实资本金。高度重视和支持金融工作，创优金融生态环境，建立政府主导的银企对接工作机制，深化与各金融机构的战略合作，确保新增贷款50亿元以上，引进1-2家股份制银行，推进保险业平稳较快发展。

（八）坚持统筹兼顾，发展社会事业。加强技术创新平台建设，力争新建科技企业孵化器1-2家。建立稳定增长的科技投入机制，抓好科技特派员工作，启动市本级科技计划项目，深入推进产学研合作，加快开发一批“高、精、尖”产品，促进科技优势转化为经济优势。进一步调整优化教育布局，加强寄宿制学校基础设施建设，逐步解决教育均衡发展不够的问题，支持发展与产业结构相适应的职业教育和民办教育，不断提高教育质量。立足于推进公共卫生服务的均等化，优化资源配置，加快基层医疗卫生机构规范化建设，加强农村卫生工作，提高城市社区卫生服务能力；大力实施重大公共卫生服务项目，有效处置各种突发公共卫生事件。抓好文化体育设施建设，满足人民群众不断增长的精神文化需要。加强网吧、娱乐、音像制品市场管理，进一步净化文化市场。深入开展全民健身运动，积极备战全省第十三届体育运动会，筹备举办市第二届体育运动会。抓好第六次人口普查。夯实计划生育基层基础工作，稳定低生育水平，下大力治理出生人口性别比偏高问题。加强国土资源管理，建立用地指标评价考核体系，保持耕地总量动态平衡。围绕发展循环经济、低碳经济，严格落实管理责任制，确保完成节能降耗考核目标。加大生态文明建设力度，着力推动省级“环保模范城”创建工作。以“塑造美好心灵，建设美好家园”为主题，深入开展精神文明创建活动，加强公民特别是未成年人思想道德教育，大力弘扬民族精神和时代精神，进一步提高市民素质，营造良好的人文环境，增强发展的软实力。加强国防动员、民防人防、民兵预备役应急能力建设，做好军队转业干部、复退军人的安置、优抚和“双拥”工作，增进军政军民团结。重视发展老龄、残疾人事业，切实保障妇女和未成年人合法权益。依法管理宗教事务，进一步做好统计、国调、审计、档案、保密、对台、史志、气象、水文、外事侨务、红十字会和无偿献血等工作。

（九）竭力改善民生，促进社会和谐。坚持把构建民生保障体系和公共安全体系作为系统工程来推进，整合各方力量，建立长效机制，努力保持社会长治久安、人民安居乐业。

一是千方百计扩大就业。实施更加积极的就业政策，一手抓扩大就业，一手抓援企稳岗。以促进返乡农民工、高校毕业生、困难和失业群体就业为重点，深入开展就业援助专项行动，落实小额担保贷款和税费减免措施，多渠道开发就业岗位，切实加强技能培训，统筹抓好城乡就业再就业；纵深推进创业带动就业，全力支持“回归创业园”建设，多途径拉动民间投资。

二是进一步加强社会保障体系建设。按照保基本、广覆盖、可持续的原则，健全完善以养老、医疗、失业保险为重点的社会保障体系，加大社会保险扩面力度，做好企业养老保险省级统筹工作，扩大被征地农民参保范围，稳步提高企业离退休人员养老金、城乡低保和优待对象补贴标准；建立大病统筹和城镇居民医疗保险门诊统筹，进一步提高新农合保障水平，稳定提高城镇居民医保报销比例。稳步推进住房公积金缴存扩面、规范管理，为改善城镇职工住房条件提供保障。新建一批廉租住房，扩大租赁补贴覆盖面，逐步解决4000户困难群众住房问题。

三是全力维护和谐安宁的社会环境。坚持标本兼治、重在治本，加强安全生产管理，重点抓好特种设备、非煤矿山、危险化学品、烟花爆竹和道路水上交通、建筑施工等行业的专项整治，严防重特大事故发生。加强应急能力建设，完善应急管理机制，健全预警监测网络和应急救援体系。加强信访维稳工作，重视解决信访突出问题，继续推行“逢四说事、有话就说”等行之有效的基层民主政治建设制度，预防和有效处置群体性事件。扎实开展“平安随州”创建活动，加强社会治安综合治理，健全完善治安防控体系，严厉打击各类违法犯罪活动，保障人民生命财产安全。

四、致力建设人民满意的政府

按照日常性工作求规范、重点性工作求突破、全局性工作争一流的要求，强化责任、规范行为、积极作为，进一步提高政府执行力和服务水平。

（一）科学决策，致力于建设法治政府。贯彻落实民主集中制原则，健全完善公众参与、专家论证和集体决策相结合的科学决策体系，建立决策风险评估机制。在认真总结“十一五”规划执行情况的基础上，集中各方面的智慧和力量，精心做好“十二五”规划编制工作。推进依法行政，加强民主法制建设，自觉接受市人大、市政协的监督，定期向人大报告工作、向政协通报情况，扎实办好议案、建议和提案。广泛认真听取各民主党派、工商联、无党派人士和人民团体的意见。重视新闻舆论和社会公众监督，支持监察、审计部门依法独立监督。进一步规范行政执法行为，加强行政复议和行政应诉工作。依法推进政府信息公开，实现市、县电子政务和电子监察系统的全覆盖；精心办好 《随州政报》和政府门户网站，畅通信息发布渠道，保障人民的知情权、参与权和监督权。深入开展普法教育，认真做好“五五”普法检查验收工作，不断加强司法援助和司法调解，引导群众依法维权。积极推进“阳光政府”建设，扩大基层民主，完善政务、厂务、村务公开，推进村民居民自治，保障人民依法直接行使民主权利。

（二）行政提速，致力于建设效能政府。牢固树立“道以通为要”的观念，再造工作流程，提高行政效率，优化政务环境。加强行政服务中心建设，按照“职能归并、集中审批、授权到位”的要求，打造“行政超市”；建立市、县两级并联办理机制，加强部门协调配合，积极构筑行政服务绿色通道，畅通市县乡村四级行政服务网络。切实增强“能力席位”意识，着力提高各级领导干部谋划发展、推动发展和群众工作、维护稳定的能力，着力提高公务员的操作能力。进一步完善目标管理，落实行政首长负责制、岗位责任制和行政问责制，保持高效率、快节奏，把政府执行力和能力建设落实到为市场主体提供方便快捷、贴心舒心的服务上来。

（三）塑造形象，致力于建设勤廉政府。坚持权为民所用、情为民所系、利为民所谋，从我做起，市政府班子成员率先垂范，各级政府班子做出表率，勤政为民不懈怠，清正廉洁不腐败。始终保持积极进取的精神状态，大力弘扬求真务实之风、干事创业之风。注重加强公务员队伍的教育、管理和监督，切实增强贯彻落实科学发展观的自觉性和坚定性。认真落实党风廉政建设责任制，坚持用制度管权、用制度管事，全面推行招投标管理 “五统一”，巩固 “四项制度”建设成果，抓好国家和省在我市开展的招投标市场综合监管体系建设试点工作，着力打造“随州模式”。牢固树立过“紧日子”的思想，艰苦奋斗，勤俭节约。切实纠正部门和行业不正之风，努力赢得全市人民的信赖与支持！

（四）办好实事，致力于建设务实政府。为人民群众办好事、办实事，是各级政府应尽的职责。在这里，我代表市人民政府郑重承诺，今年我们将集中财力，完善措施，继续为市民办好10件实事：（1）大力开展创业培训，落实小额担保贷款和税费减免扶持政策，新增5000人创业，带动城镇新增就业1.5万人，下岗失业人员再就业5000人，农村劳动力转移就业3.2万人；新增城镇居民五项基本社会保险扩面3.1万人。（2）启动实施国家基本药物目录试点，实现药物进出“零差价”收费，稳定增长城乡居民医保报销比例。（3）扎实做好曾都区全国新型农村养老保险试点工作，逐步解决农村居民老有所养问题。（4）新建城区公厕24座，改造一批破旧厕所，解决市民入厕难问题。（5）新建城区主干道6条，全部拆除原汉丹铁路的涵洞和立交桥，增设公交线路，畅通城市交通，解决市民出行难问题。（6）加快实施“校安工程”，消除校园危房32万平方米。（7）完成12个城区社区居委会标准化改造，提高保障水平，增强便民服务功能。（8）建成市残疾人康复中心，全面实施0-6岁贫困残疾儿童抢救性康复工程。（9）加大城市低收入群体安居工程建设和棚户区改造力度，投资5000万元，新建廉租住房780套，新增租赁补贴3490户。（10）大力扶持农村社会事业发展，再启动25个村的“整村推进”扶贫工作，维修改造16个乡镇综合文化站（馆），建成农家书屋300个，完成广播电视“村村通”工程212个。

各位代表！机遇与挑战同在，希望与困难并存。站在历史的新起点，面对人民的新期待，我们深感使命光荣，责任重大。让我们在中共随州市委的正确领导下，深入贯彻落实科学发展观，振奋精神，迎难而上，团结拼搏，扎实工作，奋力推进经济社会又好又快发展，以优异的成绩向建市十周年献礼！
